# Cellular HIV-1 DNA Levels in Drug Sensitive Strains Are Equivalent to Those in Drug Resistant Strains in Newly-Diagnosed Patients in Europe

**DOI:** 10.1371/journal.pone.0010976

**Published:** 2010-06-08

**Authors:** Victoria L. Demetriou, David A. M. C. van de Vijver, Ioanna Kousiappa, Claudia Balotta, Bonaventura Clotet, Zehava Grossman, Louise B. Jørgensen, Snjezana Z. Lepej, Itzchak Levy, Claus Nielsen, Dimitrios Paraskevis, Mario Poljak, Francois Roman, Lidia Ruiz, Jean-Claude Schmidt, Anne-Mieke Vandamme, Kristel Van Laethem, Jurgen Vercauteren, Leondios G. Kostrikis

**Affiliations:** 1 Department of Biological Sciences, University of Cyprus, Nicosia, Cyprus; 2 Department of Virology, Erasmus MC, University Medical Centre, Rotterdam, The Netherlands; 3 Institute of Infectious and Tropical Diseases, University of Milan, Milan, Italy; 4 IrsiCaixa Foundation, Hospital Germans Trias i Pujol, Barcelona, Spain; 5 National HIV Reference Lab, Central Virology, Public Health Laboratories, MOH Central Virology, Sheba Medical Centre, Ramat Gan, Israel; 6 Retrovirus Laboratory, Division of Diagnostic Microbiology, Department of Virology, Statens Serum Institut Copenhagen, Copenhagen, Denmark; 7 University Hospital for Infectious Diseases, Zagreb, Croatia; 8 Infectious Diseases Unit, Sheba Medical Centre, Ramat-Gan, Israel; 9 National Retrovirus Reference Centre, Department of Hygiene Epidemiology and Medical Statistics, Medical School, University of Athens, Athens, Greece; 10 Institute of Microbiology and Immunology, Faculty of Medicine, University of Ljubljana, Ljubljana, Slovenia; 11 Retrovirology Laboratory, Centre Hospitalier de Luxembourg, National Service of Infectious Diseases, Luxembourg, Luxembourg; 12 Katholieke Universiteit Leuven, Rega Institute for Medical Research, Leuven, Belgium; University of California San Francisco, United States of America

## Abstract

**Background:**

HIV-1 genotypic drug resistance is an important threat to the success of antiretroviral therapy and transmitted resistance has reached 9% prevalence in Europe. Studies have demonstrated that HIV-1 DNA load in peripheral blood mononuclear cells (PBMC) have a predictive value for disease progression, independently of CD4 counts and plasma viral load.

**Methodology/Principal Findings:**

Molecular-beacon-based real-time PCR was used to measure HIV-1 second template switch (STS) DNA in PBMC in newly-diagnosed HIV-1 patients across Europe. These patients were representative for the HIV-1 epidemic in the participating countries and were carrying either drug-resistant or sensitive viral strains. The assay design was improved from a previous version to specifically detect M-group HIV-1 and human CCR5 alleles. The findings resulted in a median of 3.32 log_10_ HIV-1 copies/10^6^ PBMC and demonstrated for the first time no correlation between cellular HIV-1 DNA load and transmitted drug-resistance. A weak association between cellular HIV-1 DNA levels with plasma viral RNA load and CD4^+^ T-cell counts was also reconfirmed. Co-receptor tropism for 91% of samples, whether or not they conferred resistance, was CCR5. A comparison of *pol* sequences derived from RNA and DNA, resulted in a high similarity between the two.

**Conclusions/Significance:**

An improved molecular-beacon-based real-time PCR assay is reported for the measurement of HIV-1 DNA in PBMC and has investigated the association between cellular HIV-1 DNA levels and transmitted resistance to antiretroviral therapy in newly-diagnosed patients from across Europe. The findings show no correlation between these two parameters, suggesting that transmitted resistance does not impact disease progression in HIV-1 infected individuals. The CCR5 co-receptor tropism predominance implies that both resistant and non-resistant strains behave similarly in early infection. Furthermore, a correlation found between RNA- and DNA-derived sequences in the *pol* region suggests that genotypic drug-resistance testing could be carried out on either template.

## Introduction

The development of antiretroviral therapy to fight HIV-1 infection has lead to a significant decrease in mortality and morbidity among infected populations. Nevertheless, the emergence of viral species resistant to drugs presents a major problem in the desired response to therapy. In the past decade, studies have been focusing on the transmission of such species in different parts of the world and it has been estimated that transmitted drug resistance occurs in about 9% of all newly diagnosed HIV-1 patients across Europe, USA and Canada [1], [2], [3], [4], [5], [6]. Also, transmitted resistance cases are frequently found to be clustered [7], [8]. This is probably explained by transmitted cases introduced before HAART became available, continuing to be transmitted today.

Integrated HIV-1 DNA in host genomic DNA acts as a latent reservoir and ensures viral persistence in spite of prolonged antiretroviral therapy [9], [10], [11], [12], [13], [14], [15]. This persistent cellular reservoir can reactivate itself and replenish viral infection, presenting itself as one of the current challenges for the control of HIV-1 infection progression [16], [17], [18]. Cellular HIV-1 DNA load is a marker associated with the viral reservoir and with the spread of the virus. Studies in patients with primary HIV-1 infection and advanced HIV-1 disease have demonstrated that early levels of HIV-1 DNA load in peripheral blood mononuclear cells (PBMC) and in CD4^+^ T-cells have a predictive value for long-term virological outcome and for disease progression, independently of CD4 counts and plasma viral RNA load [19], [20], [21], [22], [23], [24], [25], [26], [27], [28], [29], [30], [31], [32]. Many in-house protocols have been developed for the quantification of cellular HIV-1 DNA in its different forms, including end-point and real-time PCR assays [33]. However, there is still no universal or standardised way to monitor and report HIV-1 DNA quantities.

Here we present an improved method of quantification of cellular HIV-1 DNA levels. We measure the concentration of HIV-1 DNA forms which have undergone the second template switch (STS DNA) in PBMC. This detects a pool of HIV-1 forms that includes integrated and unintegrated linear dsDNA viral genomes and 1- and 2-LTR circles. A cohort of newly-diagnosed patients was studied for genotypic drug resistance, co-receptor tropism and cellular viral DNA load.

## Methods

### Ethics statement

The present study was performed as part of the EuropeHIVResistance network (www.europehivresistance.org), and ethical requirements were fulfilled according to the procedure described in the European Commission contract for EHR (project LHSP-CT-2006-518211). The procedure differs among the ten countries in the network according to national legislation. Briefly, for each participating hospital or collection centre, approval was obtained by the institutional or national medical ethical review committee and a written informed consent was obtained for each patient. In countries where a mandatory surveillance system was already established, legally no informed consent was needed. All samples were analysed anonymously and coded at national level. The names of the institutional review boards and committees are: the “Commissie voor Medische Ethiek van de faculteit Geneeskunde” associated with University Hospital Gasthuisberg, Leuven, Belgium; the “Dr. Fran Mihaljevic” University Hospital for Infectious Diseases Ethics Committee, Zagreb, Croatia; the Cyprus National Bioethics Committee, Nicosia, Cyprus; the “Videnskabsetiske Komite for VejleFyns Amter” and “Datatilsynet”, Copenhagen, Denmark; the Bioethical Committee of the Medical School, National and Kapodistrian University of Athens, Greece; the Helsinki Committee for Bioethics for Israel; the Ethics Committee for Clinical Trials, 'L. Sacco' Hospital, Milan, Italy; the “Comite National D'Ethique a la Recherche (CNER)”, Luxemburg; the Medical Ethics Committee at the Ministry of Health of Slovenia; and “Comité Ético de Investigación Clínica, Hospital Universitari Germans Trias i Pujol”, Barcelona, Spain. Approval was not required from an institutional review board for Greece as samples were made anonymous and coded on a national level.

### Sample and data collection

Included in this study were samples from 253 newly-diagnosed HIV-1 seropositive individuals from ten countries-members of the EuropeHIVResistance network: Belgium, Croatia, Cyprus, Denmark, Greece, Israel, Italy, Luxemburg, Slovenia and Spain. Sample data were obtained from the database of the E. C.-funded project EuropeHIVResistance [1], which studies the epidemiology of drug resistance among patients newly diagnosed with HIV-1 across Europe [6]. Patients were representative for the risk group and geographical distribution of the national HIV epidemic and were included within six months after diagnosis.

### PBMC isolation and genomic DNA extraction

PBMC were isolated from blood samples by one of two methods. Samples from 120 individuals were isolated from blood collected in BD Vacutainer® CPTTM (BD, Franklin Lakes, NJ, USA) tubes according to manufacturer's instructions. Samples from 71 individuals were extracted from blood collected in EDTA tubes by Ficoll density gradient centrifugation (Lymphoprep; Nycomed, Oslo, Norway). CPT™ and Ficoll density gradient separation have been found to perform equivalently in maintaining the quality and function of PBMC from HIV seropositive blood samples [34]. Genomic DNA was extracted from the isolated PBMC using the QIAmp DNA Blood Mini kit (Qiagen, Valencia, CA, USA) and eluted with 100 µl AE buffer following the manufacturer's instructions. DNA quality and quantity was evaluated for all samples by UV spectrophotometry using NanoDrop ND-1000 (NanoDrop Technologies, Wilmington, DE, USA).

### Genomic DNA extraction from whole blood

DNA from samples from 62 individuals was extracted from whole blood collected in EDTA tubes. For 15 samples, DNA was isolated from 0.5 ml whole blood using the QIAmp DNA Blood Mini kit (Qiagen) and eluted in 100 µl AE buffer. For 47 samples, DNA was isolated from 0.3 ml whole blood using the High Pure PCR Template Preparation kit (Roche Molecular Diagnostics, Manheim, Germany), and eluted in 50 µl dH_2_O. Due to the production of irregular signals from the real-time PCR, the latter samples were cleaned using the cleaning and elution steps of the QIAmp DNA Blood Mini kit (Qiagen), and eluted in 100 µl AE buffer.

### HIV-1 subtyping and determination of drug-resistance

Samples were amplified and sequenced in the *pol* region as described previously [35]. Subtype was determined by uploading the sequences individually into the REGA HIV-1 & 2 Automated Subtyping Tool v2.0 [36]. This was confirmed with phylogenetic analysis by constructing a Neighbour-Joining tree [37] using the Kimura-2-parameter distance estimation approach [38] with MEGA v4 [39]. The reliability of clustering was evaluated using bootstrap analysis with 1,000 replicates [40], where bootstrap values above 70 were considered significant for subtype assignment. Standard reference sequences used in the REGA HIV subtyping tool [36] were downloaded from the website (http://www.bioafrica.net/rega-genotype/html/subtypinghiv.html).

Drug resistance in the samples was determined by examination of the PR/RT sequence of each for mutations known to confer resistance to protease and reverse transcriptase inhibitors. This was done by manually examining the sequences and verified automatically using the Stanford drug-resistance algorithm [41], [42], as described previously [35]. TDRM have been defined as the presence of at least one of the following mutations in protease: 30N, 46I/L, 48V, 50L/V, 82A/F/T/S, 84A/C/V, 90M; or RT: 41L, 44D, 62V, 65R, 67N, 69D/insert, 70R, 74V, 75I, 77L, 100I, 103N, 106A/M, 108I, 115F, 116Y, 151M, 181C/I, 184I/V, 188C/H/L, 190A/S, 210W, 215Y/F, 215 revertants A/C/D/E/G/H/I/L/N/S/V, 219Q/E, 225H, 230L, 236L [43].

### Comparison of RNA- and DNA-associated *pol* sequences

HIV-1 *pol* nucleotide sequences covering the PR/RT region derived from plasma viral RNA and cellular DNA were compared to determine any correlation between the two. The sequences were examined in the positions known to confer resistance to antiretroviral drugs, in order to establish whether the genotypic drug resistance testing on the two types of sequences resulted in different data.

### Determination of co-receptor tropism by HIV-1 V3-loop amplification and sequencing

All samples were amplified by PCR in the V3-loop region with a nested PCR using extracted genomic DNA from the PBMC samples. The primary PCR was performed using 3 µl DNA in a 50 µl reaction using Platinum® PCR SuperMix (Invitrogen, Carlsbad, CA, USA) with 20 pmol each of the outer forward (5′-ATGGGATCAAAGCCTAAAGCCATGTG-3′, positions 6557-6582) and reverse (5′- AGTGCTTCCTGCTGCTCCCAAGAACCCAAG-3′, positions 7811-7782) primers. The cycling conditions were one cycle at 94°C for 2 min, 40 cycles at 94°C for 20 s, 55°C for 30 s, 72°C for 30 s, and a final step at 72°C for 7 min. A nested PCR was performed using 3 µl of the primary PCR product with 20 pmol each of the inner forward (5′-CCAATTCCCATACATTATTGTGCCC-3′, positions 6858-6882) and reverse (5′-TTACAGTAGAAAAATTCCCCTCCACAATTAAA-3′, positions 7381-7350) primers in a 50 µl reaction, with the same cycling conditions as above, but with an annealing temperature of 52°C. Cycle sequencing PCR was performed on purified amplicons bidirectionally using the inner forward and reverse amplification primers by means of the BigDye® Terminator system v3.1 (Applied Biosystems, Foster City, CA, USA). The products were sequenced directly on the ABI 3300 Genetic Analyser (Applied Biosystems). The resulting readings were analyzed with the Sequencing Analysis Software v5.2 (Applied Biosystems). To determine the co-receptor tropism of the samples, sequences were uploaded into the geno2pheno [44] and WebPSSM [45], [46] automated bioinformatics tools, using default settings.

### Cellular HIV-1 STS load in newly-diagnosed, drug-naïve samples

**Figure 1:**
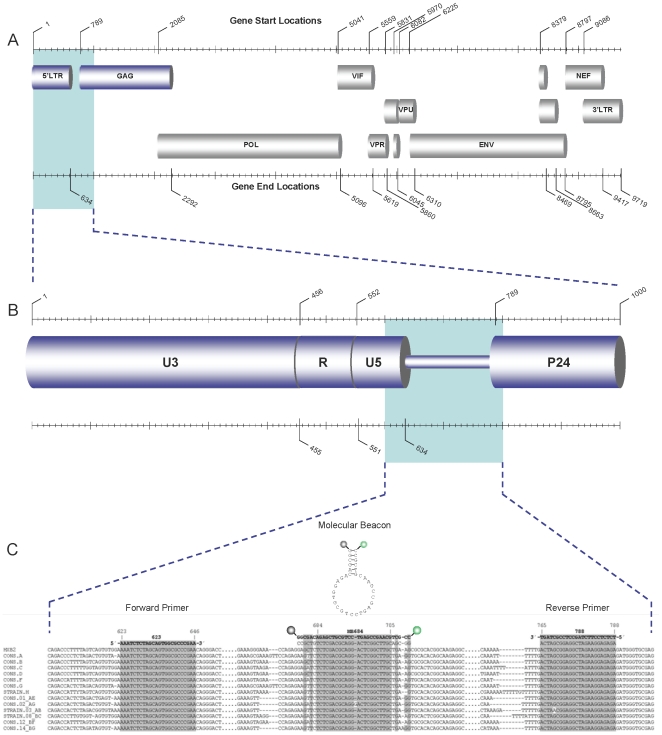
Schematic diagram of the HIV-1 assay design. Schematic diagram of the HIV-1 genome (A) and the region targeted in this study (B). Below (C), a sequence alignment of the HXB2 strain and consensus sequences of the most widely distributed subtypes in the M group from nucleotide position 600 to 800 according to the numbering of strain HXB2, constructed from sequences available on the Los Alamos HIV sequence database (labelled as CONS). For each of the subtypes H, CRF03_AB and CRF08_BC only one sequence was available in this region and a consensus could not be made (labelled as STRAIN). No sequences were available for subtypes J and K in this region. The sequences and names of the primers and molecular beacon used in this study are seen in bold above the alignment. Directly below the beacon and reverse primer sequence is the complementary sequence corresponding to the viral positive strand. Above the beacon sequence is a schematic representation of the beacon in its closed conformation (hairpin loop). The beacon probe is labelled with a fluorophore (FAM) on the 5′end (seen as a green circle) and a DABCYL quencher on the 3′end (seen as a dark grey circle). The primer and molecular beacon sequences and their exact targets in the alignments are highlighted in grey. Dots in the sequences represent unseen regions of the alignment and dashes represent gaps in the sequences produced by the alignment.

To uniquely detect HIV-1 DNA structures that have completed the two template switches, a molecular-beacon-based real-time PCR assay reported previously [20] has been improved for better compatibility and specificity to the target, by using the general method of quantifying single sequences with nucleotide-specific molecular beacons [47] and real-time PCR [48] as previously described [20], [49]. The assay uses primers that amplify a smaller region, from the end of U5 to just before the gag sequence, from position 623 to 788 (numbers according to sequence HXB2). The PCR primers and the target recognition sequence of the molecular beacon were designed to hybridize on conserved regions from all the genetic subtypes within the M group based on a comprehensive DNA sequence alignment from published HIV-1 sequences (Table 1 and Figure 1). This assay detects only HIV-1 DNA structures that have undergone both single stranded DNA template switches, including both unintegrated and integrated linear viral genomes. To confirm the specificity of the design, amplicons were sequenced and found to contain the correct region. To assess the thermodynamic characteristics, the quality and the purity of the molecular beacon, a melting curve analysis was performed by monitoring the fluorescence thermal transition profiles using the 7900HT Real-Time PCR System (Applied Biosystems, Foster City, California, USA). The cycling parameters were as follows: 1 cycle for 2 min at 95°C followed by 50 cycles each consisting of the data collection step for 30 s and a second step for 10 s, starting at 80°C with an auto-incrementation of −1°C per half-minute cycle until 31°C were reached. The reaction consisted of a 25 µl mixture containing 1X Platinum® Quantitative PCR Supermix-UDG (Invitrogen, Carlsbad, CA), 5.7 pmol of the beacon probe with or without 100 pmol of a perfectly complementary single-stranded oligonucleotide target. Changes in fluorescence were measured at 490 nm and the data collected at each temperature interval were plotted to form these thermal denaturation profiles and determine the optimal annealing temperature for the real-time PCR reaction.

**Table 1:** Molecular beacons and primers used in the real-time PCR assay.

Name	Oligonucleotide	Sequence (5′-3′) a	Position b	Reference
**HIV-1**				
MB684	Molecular beacon	FAM- CCGCTGCAAGCCGAGTCCTGCGTCGAGACAGCGG -Dabcyl	684–705	Modified from [20]
623	Forward primer	AAATCTCTAGCAGTGGCGCCCGAA	623–646	Modified from [20]
788	Reverse primer	TCTCTCCTTCTAGCCTCCGCTAGT	765–788	Modified from [20]
**CCR5**				
LK155	Molecular beacon	TET- GCGCCTATGACAAGCAGCGGCAGGAGGCGC -Dabcyl	623–641	Kostrikis et al., unpublished data
LK46	Forward primer	GCTGTGTTTGCGTCTCTCCCAGGA	478–501	Kostrikis et al., unpublished data
LK47.new	Reverse primer	CACAGCCCTGTGCCTCTTCTTCTCA	690–713	Kostrikis et al., unpublished data

^*a*^FAM, fluorescein; TET, Tetrachloro-6′-carbofluorescein; Dabcyl, 4-(4′-dimethylamino phenylazo)benzoic acid; underlined sequences indicate the complementary sequences forming the molecular beacon hairpin structures.

^*b*^Positions correspond to the GenBank sequences K03455 and U83326.1 for HIV-1 and CCR5, respectively. For molecular beacons, the nucleotide positions correspond to the target recognition sequences (non-underlined sequences).

### Assay design for quantification of human *CCR5* alleles

To quantify the number of cells in the input DNA, a molecular-beacon-based real-time PCR assay was used for the detection of *CCR5* alleles. The design was based on a previously published protocol [20] and is an improved version for the quantification of a region of the human *CCR5* gene adjacent to the *Δ32* deletion, which exists at two copies per cell (L. G. Kostrikis, unpublished data), allowing the quantification of genomic equivalents in a given sample. The PCR primers and the target recognition sequence of the molecular beacon are listed in Table 1. A thermal denaturation curve was constructed as described above, with 6.5 pmol of the CCR5-specific molecular beacon.

### CCR5 and HIV-1 standards and standard curves

Cloned plasmids containing within them the targeted HIV-1 DNA and CCR5 amplicons of the real-time PCR assays were used as external quantification standards in the experiments. The amplicons were cloned into plasmids (TOPO TA Cloning® Kit, pCR 4-TOPO vector, Invitrogen, Carlsbad, CA, USA) following PCR with the primers seen in Table 1. Five clones of each were selected and sequenced on the ABI 3130 Genetic Analyzer (Applied Biosystems, Foster City, CA, USA) using the vector primers available from the cloning kit (Invitrogen) as sequencing primers, to ensure correct insertion of the amplicons into the plasmids. Concentrations were measured by UV absorbance using a NanoDrop 1000 spectrophotometer (NanoDrop Technologies, Wilmington, DE, USA) and the copy number per unit volume was calculated. Tenfold serial dilutions of the purified plasmids of known molar concentrations were used as templates to generate standard curves in the selective amplification assays. Standard curves were made by plotting the threshold cycle of known CCR5 and HIV-1 copies per reaction. The slope and correlation coefficient of each standard curve were calculated based on the median threshold cycle (C_T_) values measured for replicates of each dilution point ranging from 10^6^ to 10^1^ DNA templates. The PCR efficiency, E, corresponding to the experimentally derived dynamic range was computed as (10^−1/s^ − 1)×100, where s is the slope of the standard curve generated.

For the real-time PCR reaction, each 25 µl reaction mixture contained 5 µl of extracted genomic DNA, 20 pmol each of forward and reverse primer, 1X Platinum® qPCR Supermix-UDG (Invitrogen, Carlsbad, CA, USA). For the CCR5 reaction, 6.5 pmol of beacon LK155 were used and in the HIV-1 reaction, 5.7 pmol of beacon MB684. The cycling conditions were the same for the two reactions and are as follows: one cycle of denaturation (95°C for 10 min), followed by 50 cycles of amplification (denaturation at 95°C for 15 s, annealing and data collection at 55°C for 30 s, and polymerization at 72°C for 30 s), performed on the 7900HT Real-Time PCR System (Applied Biosystems, Foster City, CA, USA). During the data-collection stage of each cycle, fluorescence emission was recorded at 490 nm.

### Quantification of cellular HIV-1 STS DNA in newly-diagnosed, drug-naïve samples

Two uniplex molecular beacon-based real-time assays were carried out in triplicate for absolute quantification of CCR5 copies and HIV-1 STS DNA copies in clinical samples, as described above for the standards. In each experiment, standard curves for HIV-1 and human CCR5 templates were also run in triplicate by using six serial dilutions, ranging from 10^6^ to 10^1^ copies, along with no-template negative controls. Each experiment was assessed by the quality of its standard curves, i.e. slope, R^2^-value and y-intercept. CCR5 and HIV-1 copies in each sample were quantified by use of the obtained threshold values from the samples and the corresponding standard curve, constructed from multiple measurements of standards. In each sample, the number of cells was quantified as one cell per two CCR5 copies, and the HIV-1 STS DNA levels were calculated per 10^6^ PBMC. The cellular viral load of a sample is, therefore, the number of HIV-1 copies per million cells.

For samples whose DNA was extracted from whole blood, HIV-1 STS DNA was quantified per ml blood and CCR5 measurements were not made, as whole blood contains many cell types, and not only PBMC. Hence, HIV-1 STS DNA load in samples obtained from whole blood are not comparable to samples extracted from PBMC.

### Statistical analysis

Viral loads were log-transformed. The cellular viral load was compared between patients infected with or without a drug resistant virus using the Mann-Whitney test. Categorical variables were compared using chi-square test. To determine whether a differential cellular viral load was observed between subtypes a Kruskal-Wallis test was performed. The 95% confidence interval of the prevalence of transmitted resistance was calculated using the Wilson score interval. The association between the plasma viral load and the cellular viral load was determined using Pearson's correlation coefficient.

## Results

### Description of the population

Available were 191 PBMC samples from HIV-1 infected individuals from eight country-members of the EuropeHIVResistance network. A total of 30 samples were excluded from the analysis due to failure of PCR in the *pol* region (n = 18), or a polymorphic nature of the sequence (n = 12), and drug-resistance could therefore not be determined. Table 2 lists the baseline characteristics of the 161 study subjects that were included in the analysis. The median age of the participants was 34 (interquartile range IQR 29–42) and they were predominantly male (72.0%), a fact also reflected by a large proportion of MSM (50.3%). The median log_10_ plasma RNA load in the samples studies was 4.43 copies per ml (IQR 3.87–5.14) and the median CD4^+^ T-cell count was 423 per ml (IQR 255.3–578.5).

**Table 2:** Characteristics of study subjects.

Characteristics a	Subjects N = 161 (%)
**Sampling country**	
Belgium	18 (11.2)
Croatia	5 (3.1)
Cyprus	40 (24.8)
Greece	8 (5.0)
Israel	18 (11.2)
Luxembourg	42 (26.1)
Slovenia	21 (13.0)
Spain	9 (5.6)
**Year of sampling**	
2002	6 (3.7)
2003	31 (19.3)
2004	12 (7.5)
2005	38 (23.6)
2006	27 (16.8)
2007	17 (10.6)
2008	30 (18.6)
**Resistance characteristics**	
Resistant to PI	4 (2.5)
Resistant to RTI	15 (9.3)
No resistance	142 (88.2)
**Gender**	
Male	116 (72.0)
Female	28 (17.4)
Unknown	17 (10.6)
**Risk group**	
MSM	81 (50.3)
Heterosexual	43 (26.7)
IVDU	4 (0.2)
Unknown	33 (18.2)
**Age (median, IQR)** b	34 (29–41.5)
**Log_10_ cellular HIV-1 DNA load (median, IQR)**	3.32 (2.92–3.75)
**Log_10_ plasma RNA load (median, IQR)**	4.43 (3.87–5.14)
**CD4^+^ count (median, IQR)**	423 (255.3–578.5)
**Subtype**	
A	23 (14.3)
B	103 (64.0)
C	14 (8.7)
D	1 (0.6)
F1	1 (0.6)
G	7 (4.3)
CRF02_AG	6 (3.7)
CRF01_AE	4 (2.5)
C/H/K	1 (0.6)
CRF02_B	1 (0.6)
**Tropism**	
CCR5	147 (91.3)
CXCR4	5 (3.1)
CXCR4/CCR5	7 (4.3)
N/A	2 (1.2)

^*a*^IQR, interquartile range.

^*b*^Data available for 130 patients.

### HIV-1 subtyping,drug-resistance and co-receptor tropism

**Figure 2:**
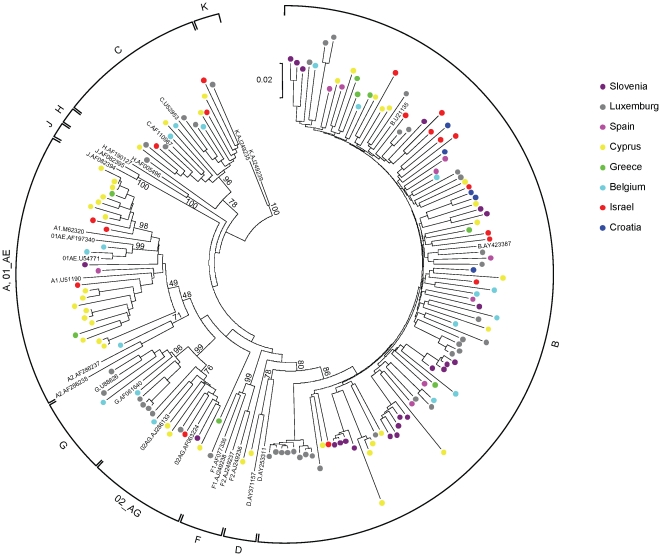
Phylogenetic tree of the samples used in the study. Neighbour-joining phylogenetic tree for the 161 HIV-1 *pol* sequences (1233 nucleotides, corresponding to positions 2253–3485 of HXB2). Trees were constructed using 48 representative reference sequences from 9 known subtypes (A through F) and 13 known recombinant strains. The sequences determined in the study are colour-coded, with colours corresponding to the country of sampling: Belgium (light blue), Croatia (dark blue), Cyprus (yellow), Greece (green), Israel (red), Luxemburg (grey), Slovenia (purple), Spain (pink). The divergence between any two sequences is obtained by summing the branch length, using the scale at the lower left of each tree. The numbers indicated at the subtype-determining nodes are percentage bootstrap support for 1,000 replicates.

HIV-1 subtype was determined using the *pol* sequences obtained above using REGA [36] and by phylogenetic analysis. The results are listed in Table 2 and the phylogenetic tree is seen in Figure 2. The most frequently observed subtype was B (64%), followed by A (14.3%) and C (8.7%). Interestingly, cases of complex recombinants (C/H/K, CRF2/B) were also present in the dataset. Sequences of all samples were examined for known resistance mutations to available protease and reverse transcriptase inhibitors. Transmitted resistance in the form of major drug resistant mutations was observed in 11.7% (95% confidence interval 7.6 to 17.6%) of the samples.

All samples were sequenced in the V3-loop and tropism was determined by the geno2pheno and WebPSSM automated bioinformatics tools. The majority of samples were CCR5-tropic (147 samples, 91.3%), and the rest were CXCR4-tropic (5 samples, 3.1%) or a mixture of CCR5 and CXCR4 (7 samples, 4.3%). Two samples (1.2%) could not be determined due to the polymorphic nature of the V3-loop sequence. None of the five CXCR4-tropic samples displayed genotypic drug-resistance, but three of the mixed-trpism samples did. The rest of the drug-resistant samples were purely CCR5-tropic.

### Comparison of RNA- and DNA-associated *pol* sequences

Known positions conferring resistance to antiretroviral therapy from HIV-1 *pol* nucleotide sequences covering the PR/RT region derived from plasma viral RNA and cellular DNA were compared for each sample, to determine any correlation between the two. The comparison showed that there is a high similarity between the two types of sequences.

### Characteristics of the real-time PCR assay for quantification of HIV-1 STS DNA in PBMC

**Figure 3:**
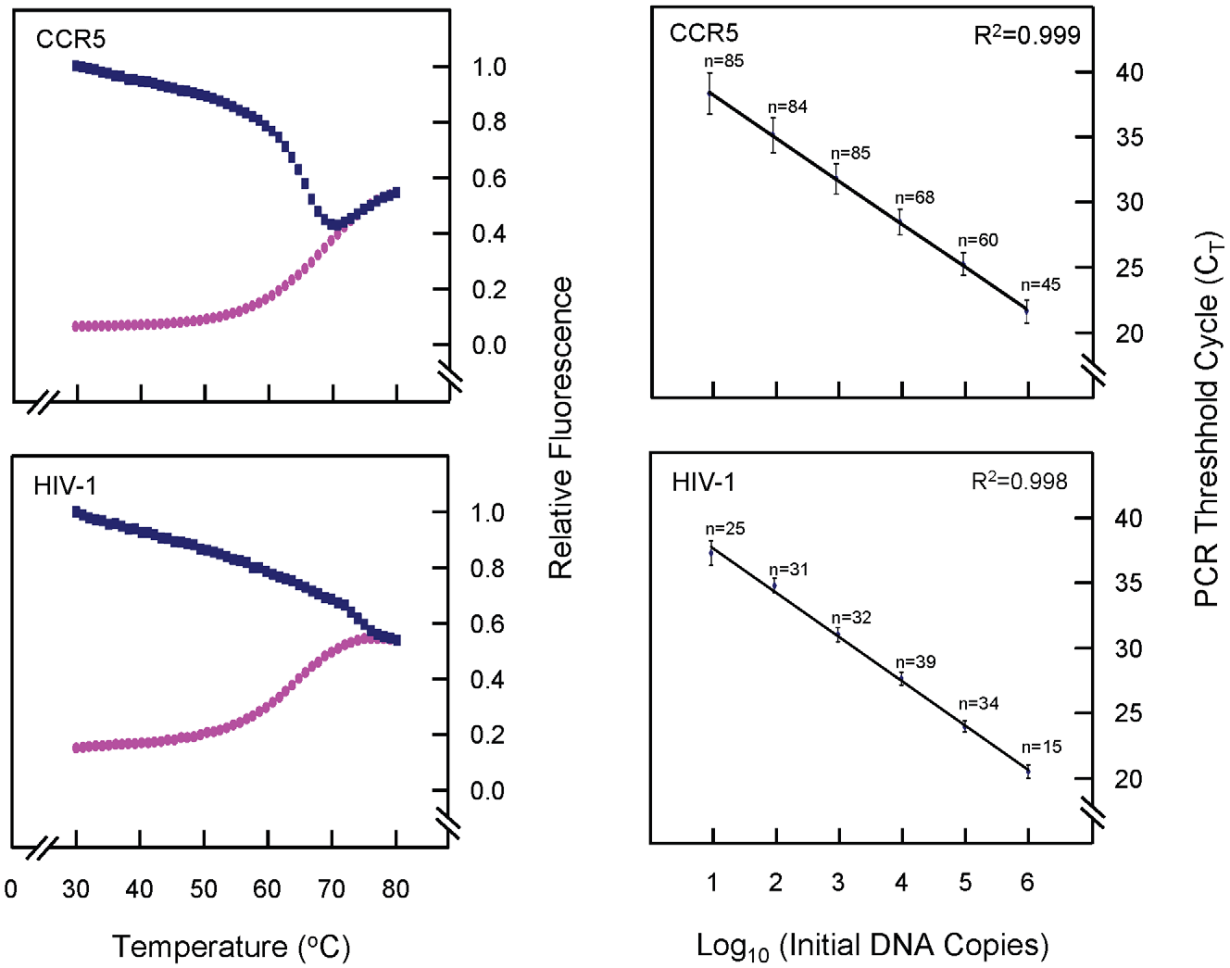
Thermal denaturation curves of the molecular beacons and standard curves for quantification. On the left are normalised fluorescence thermal transitions of molecular beacon (pink circles) and beacon-target complexes (blue squares) designed for the detection of the human *CCR5* gene and HIV-1 STS DNA. Fluorescence signals differ between the complementary molecular-beacon-target hybrids and the non-hybridized molecular beacon at temperatures below 60°C. At higher temperatures, secondary structures within and between oligonucleotides are denatured and the beacon is free in solution in a dynamic open conformation. The temperature selected (55°C) for hybridisation in the standard PCR reactions allows optimal resolution of the fluorescence signal. On the right are standard curves for human CCR5 and HIV-1 STS DNA templates used in the real-time PCR assays for quantifying HIV-1 STS DNA in human PBMC. Six serial dilutions ranging from 10^6^ to 10^1^ DNA templates were made for each DNA standard, and all standard dilutions were measured by real-time PCR using nucleotide sequence-specific molecular beacons. Median C_T_ values (± standard deviations) were measured for a number of replicates for each dilution point, indicated on the standard curves. The correlation coefficients (R^2^) of the standard curves were >0.99, and the PCR efficiencies were >99%.

Thermal denaturation profiles of both molecular beacons were constructed as described in Materials and Methods and are seen in Figure 3. The optimum concentrations of the molecular beacons were determined, as was the optimum temperature for annealing and data collection in the PCR reactions (55°C). The HIV-1 STS DNA and CCR5 assays' specificity was established by accurate detection of 10 DNA copies with a 6-log_10_ linear dynamic range. The slopes of the standard curves (Figure 3) were -3.3 cycles/log_10_ DNA templates for both assays, corresponding to PCR efficiencies of >99%. The capability of the STS DNA assay to detect HIV-1 strains within the M group was evaluated by using DNA extracted from primary PBMC samples isolated from patients infected with HIV-1 strains from genetic subtypes A, B, C, D, F1 and G and recombinant strains A/G, C/H/K, CRF01_AE, CRF02_AG and CRF02/B. The specificity of the STS DNA assay against human genomic DNA were examined using DNA extracted from individuals not infected with HIV-1; the results were negative, with no detection after 50 PCR cycles (data not shown).

### Cellular HIV-1 STS load in newly-diagnosed drug-naïve samples

**Figure 4:**
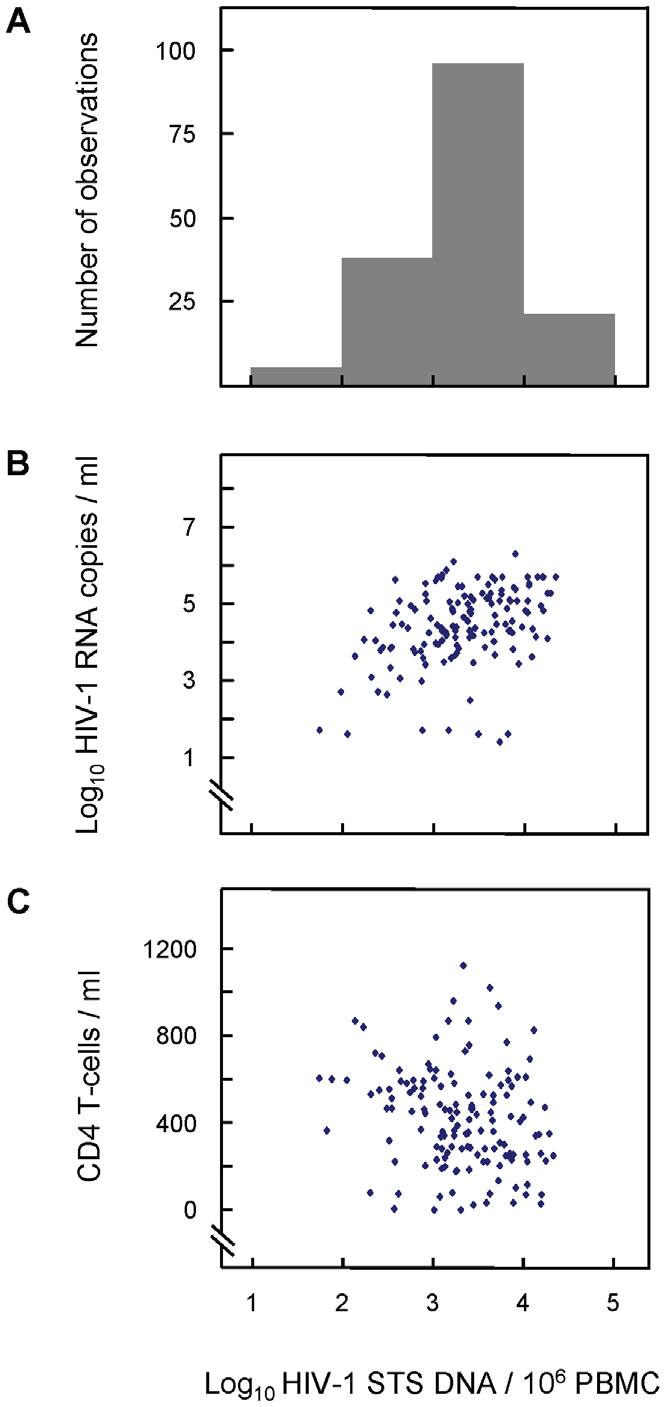
Cellular HIV-1 STS DNA load against plasma HIV-1 RNA load and CD4^+^ T-cell count. Distribution of cellular HIV-1 STS DNA load (A), plasma HIV-1 RNA load (B) and CD4^+^ T-cell counts (C) among 161, 109 and 161 newly-diagnosed individuals, respectively. The results indicate a weak correlation between STS DNA levels and plasma RNA levels (R^2^ = 0.20; *P* = 0.001) and a very weak correlation between STS DNA levels and CD4^+^ counts (R^2^ = 0.04; *P*<0.001).

The DNA samples extracted from whole blood produced very low proportion of detectable HIV-1 DNA levels (data not shown), due to the fact that whole blood contains many cell types, and were not used in the analysis. The values of cellular HIV-1 STS DNA loads in 191 samples extracted from PBMC of newly-diagnosed individuals from eight European countries were measured using molecular beacon-based real-time PCR assays quantifying HIV-1 STS DNA and human CCR5 copies per sample. The cellular HIV-1 STS DNA load was calculated as HIV-1 STS DNA copies per million cells and the median log_10_ value measured was 3.32 (IQR 2.92–3.75 log_10_ copies/10^6^ PBMC) (Figure 4A). These values were investigated according to the various known parameters of the samples.

The relationship between the cellular HIV-1 DNA loads measured in this study and other clinical parameters and characteristics of the samples at the time of sampling were also investigated. The results show that the cellular HIV-1 STS levels have a weak correlation with the corresponding plasma RNA levels (Pearson correlation coefficient R^2^ = 0.20; *P* = 0.001) and a very weak correlation with CD4^+^ T-cell counts (Pearson correlation coefficient R^2^ = 0.04; *P* = 0.013) (Figure 4B and C), confirming previously acquired data [20]. For statistical reasons, the analysis regarding subtypes was limited to the three most frequently observed subtypes, A, B and C. The median cellular HIV-1 DNA load was different in the three groups (2.91, 3.26 and 3.56 log_10_ copies/10^6^ PBMC respectively), but this difference did not reach statistical significance (*P* = 0.40).

**Figure 5:**
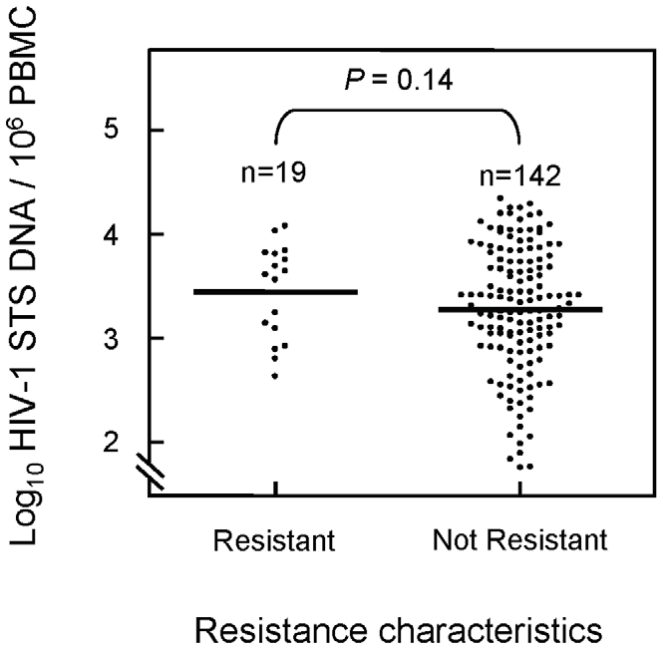
Cellular HIV-1 DNA load in drug-resistant and non-resistant samples. Dot plot of log_10_ cellular HIV-1 STS DNA load per million PBMC among 161 newly-diagnosed individuals with major drug-resistant mutations (n = 19) and with no major drug-resistant mutations (n = 142). The results indicate a trend towards higher cellular HIV-1 STS DNA load (*P* = 0.14) in resistant samples (median 3.64, IQR 2.63–4.25) compared to non-resistant samples (median 3.27, IQR 2.90–3.73).

Figure 5 shows the comparison of the cellular HIV-1 DNA loads for patients infected with drug-resistant or non-resistant strains of the virus. The results indicate a trend towards a higher cellular HIV-1 DNA load in patients with transmitted drug-resistant strains of the virus (3.64 log_10_ copies/10^6^ PBMC, IQR 2.63–4.25) than in patients infected with wild-type strains (3.27 log_10_ copies/10^6^ PBMC, IQR 2.90–3.73). This difference did, however, not reach statistical significance (*P* = 0.14). The possibility of higher cellular viral loads being explained by more recent infections in the resistance group was investigated but the proportions of recent infections in patients with or without evidence of transmitted resistant strains was (3/22) 13.6% and (10/66) 15.2% respectively (*P* = 1.00), meaning this was not the case.

## Discussion

In this study the association between cellular HIV-1 DNA load and transmitted drug resistance was examined for the first time, using an improved molecular beacon-based real-time PCR assay for quantification of HIV-1 STS DNA and human *CCR5* alleles. The median cellular HIV-1 STS DNA load calculated in this study is 3.32 log_10_ copies per 10^6^ PBMC. The results of this study indicate no statistically significant difference in cellular HIV-1 DNA load in patients with transmitted drug-resistant strains of the virus compared to that of patients infected with wild-type strains. The cellular viral DNA levels found here are higher than those quantified with the previous version of the assay [20] (*P*<0.001) due to the improvement of the specificity of the CCR5 components. This study includes a significant number of patients from Israel and countries distributed throughout Europe. The criteria of inclusion, the well-defined datasets and the consistent sampling method are encouraging parameters supporting the strength of this report. The results found are comparable to those found in previous studies of cellular HIV-1 DNA quantification in treatment-naïve patients where levels of 2.85–3.2 log_10_ copies/10^6^ PBMC were reported [19], [21], [29], [50], [51]. The findings of this study also indicate a weak association between the cellular HIV-1 DNA counts and viral RNA levels or CD4^+^ T-cell counts, in agreement with previous findings [20], [50], [52]. The three main subtypes also appeared not to have an impact on cellular viral DNA load.

Positions known to confer resistance to antiretroviral drugs in plasma RNA- and cellular DNA-derived nucleotide sequences covering the PR/RT region were compared for each patient in order to asses the level of similarity between them. Significantly, a high similarity was found between the two, suggesting that genotypic drug-resistance testing could be carried out on either starting material.

The co-receptor tropism of all samples was determined from the cellular DNA and it was found that 91% of the samples were CCR5-tropic, as expected for early infections, 3% were CXCR4-tropic and 4% were a mixture of the two types. Hence, it appears that, the existence of transmitted drug resistance mutations does not affect the co-receptor tropism of the virus. This data suggests that resistant and susceptible viral species behave similarly in early infection.

A limitation to the study is the possibility of the occurrence of reversion of viral strains to wild-type in those samples considered non-resistant, as this may play a role on cellular viral DNA load as minority species in the viral population. Nonetheless, reversion only appears to occur to a limited extent [53]. Another factor that may influence such findings is the fact that known mutations that confer resistance to antiretroviral drugs are heterogeneous and may have differential impact of resistance. The study of different TDRM individually, however, is not possible with the datasets currently available. Also, mutations associated with reduced viral fitness, and therefore lower viral loads, are not found frequently in transmitted resistant strains due to the fact that the level of plasma viral load is a key factor in the transmission of the virus [54]. Another point is the possibility of the inclusion of patients who are in the acute phase of infection at the time of sampling, who may account for values on the high end of the range of HIV cellular DNA levels obtained in this study.

The results show for the first time that there is no association between cellular viral DNA levels and the existence of transmitted drug resistant mutations in newly-diagnosed patients. Cellular HIV-1 DNA load has been shown to be associated with disease progression [19], [20], [21], [23], [24], [26], [29], [30], [32] and transmitted drug resistance does not currently have an impact on the time of treatment initiation. From this data, it is implied that transmitted drug resistance is not associated with disease progression.

Overall, this report presents an improved methodology of cellular HIV-1 DNA quantification with molecular beacon-based real-time PCR. The patients included in the study were newly-diagnosed individuals from Israel and all over Europe. The data show a similar co-receptor tropism for resistant and non-resistant viral species, implying that both behave in a similar manner in early infection. Significantly, a high correlation was found between RNA- and DNA-associated PR/RT sequencing indicating that genotypic drug resistance testing could be carried out on either type of sample. It was found that no correlation exists between cellular HIV-1 DNA levels and transmitted drug resistance, indicating that the latter may not be associated with disease progression and should continue not having an impact on the time to initiate treatment in patients.
